# Addressing challenges in the production and analysis of illumina sequencing data

**DOI:** 10.1186/1471-2164-12-382

**Published:** 2011-07-29

**Authors:** Martin Kircher, Patricia Heyn, Janet Kelso

**Affiliations:** 1 Max Planck Institute for Evolutionary Anthropology, Department of Evolutionary Genetics Deutscher Platz 6 04103 Leipzig, Germany; 2 Max Planck Institute of Molecular Cell Biology and Genetics Pfotenhauerstrasse, 108 01307 Dresden, Germany

## Abstract

Advances in DNA sequencing technologies have made it possible to generate large amounts of sequence data very rapidly and at substantially lower cost than capillary sequencing. These new technologies have specific characteristics and limitations that require either consideration during project design, or which must be addressed during data analysis. Specialist skills, both at the laboratory and the computational stages of project design and analysis, are crucial to the generation of high quality data from these new platforms. The Illumina sequencers (including the Genome Analyzers I/II/IIe/IIx and the new HiScan and HiSeq) represent a widely used platform providing parallel readout of several hundred million immobilized sequences using fluorescent-dye reversible-terminator chemistry. Sequencing library quality, sample handling, instrument settings and sequencing chemistry have a strong impact on sequencing run quality. The presence of adapter chimeras and adapter sequences at the end of short-insert molecules, as well as increased error rates and short read lengths complicate many computational analyses. We discuss here some of the factors that influence the frequency and severity of these problems and provide solutions for circumventing these. Further, we present a set of general principles for good analysis practice that enable problems with sequencing runs to be identified and dealt with.

## Background

Recent advances in DNA sequencing have changed the field of genomics making it possible to generate gigabases of genome and transcriptome sequence data at substantially lower cost than was possible just ten years ago http://www.genome.gov/sequencingcosts/. The relative affordability of these high-throughput sequencers and the potential to generate large amounts of sequence data at lower cost means that scientists outside of traditional sequencing facilities are now faced with the challenges associated with design of large-scale projects and analysis of the data generated. This poses significant challenges for many groups since the inherent limitations of these platforms, and particular artifacts associated with sequences generated on these platforms, need to be understood and dealt with at various stages of the project including planning, sample preparation, run processing and downstream analyses.

We present here an analysis of challenges encountered in using the Illumina sequencing instruments. A thorough description of the Solexa/Illumina sequencing technology as well as a comparison to other platforms is available elsewhere [1-6]. We revisit here only the aspects relevant for project design and data analysis.

**Figure 1:**
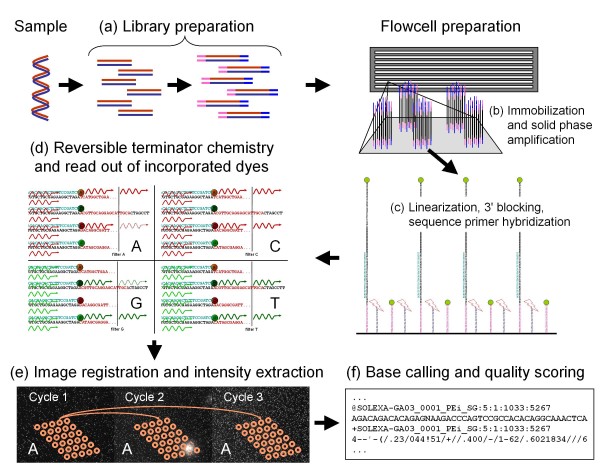
**Illumina sample preparation and sequencing**. Illumina sequencing requires that a DNA sample (a) is converted into special sequencing libraries. This can be achieved by shearing DNA to a designated size and adding specific adapter sequences on both ends of the DNA molecules (b). These adapters allow molecules to be amplified and immobilized in one or more channels of an 8-channel flow cell (c). Immobilization and solid-phase amplification create randomly scattered clusters, consisting of a few thousand copies of the original molecule in very close proximity to each other. One of the DNA strands is removed to obtain single stranded, identically oriented copies, 3' ends of the DNA are blocked and a sequencing primer hybridized on the adapter sequences. Afterwards, the reversible terminator chemistry is performed (d). Here, four differently labeled nucleotides are provided and used for extension of the primers by DNA polymerases. The polymerase reaction terminates after the first base incorporation since the nucleotides used are not only labeled, but also 3'-blocked. After washing away free nucleotides, the nucleotides incorporated are readout by piece-wise imaging of the flow cell. Then, the terminator and fluorophore are removed and another incorporation cycle started. The four images are overlaid (registered) and light intensities extracted for each cluster and cycle using a cluster position template obtained from the first instrument cycles (e). Resulting intensity files serve as input for base calling, the conversion of intensity values into bases and quality scores (f).

Figure 1 shows the steps from the DNA sample preparation to sequence read outs with quality scores. Independent of the actual application, Solexa/Illumina sequencing requires that the molecules to be determined are converted into special sequencing libraries. This is achieved by adding specific adapter sequences on both ends of fragmented DNA molecules; allowing molecules to be amplified, immobilized and primed for sequencing. For sequencing, typically double-stranded DNA libraries are provided and melted using sodium hydroxide to obtain single stranded molecules. These are then immobilized (hybridization and amplification on a solid phase; bridge amplification [1,7]) in one or more channels of an 8-channel flow cell. The immobilization and subsequent bridge-amplification creates randomly scattered clusters, consisting of more than one thousand copies of the original sequence in very close proximity to each other. After the bridge amplification step, cluster molecules are largely double stranded. One of the strands then has to be removed to obtain single stranded, identically oriented copies of the starting molecule. This is achieved by selective cleavage of base modifications of oligonucleotides on the flowcell. After the free 3' ends of the DNA have been blocked, the copies can be sequenced by hybridizing a sequencing primer onto the adapter sequences and starting the reversible terminator chemistry. Here, four differently labeled nucleotides are provided and used for extension of the sequencing primers by DNA polymerases. The DNA polymerase reaction terminates after the first base incorporation since the nucleotides used are not only labeled, but also 3'-blocked (i.e. they carry a terminator group at the third carbon atom of the sugar, which prevents further extension). After free nucleotides are washed away, the nucleotides being incorporated are read by capturing the light signal of the fluorophore labels after laser excitation. Imaging of the flow cell is carried out in so-called tiles which are the units in which the flowcell is imaged and data processed. The terminator and fluorophore are then removed and another incorporation cycle started [1].

Initially, the number of sequencing cycles, and thereby the length of the sequence reads, was limited to 26 cycles because of steeply increasing sequencing error. Between 2008 and 2010 there were several technical updates to the Genome Analyzer (GA) platform including improvements in mechanics, chemistry and software. Even though sequencing error still increases with each cycle, up to 150 sequencing cycles are currently performed with reasonable error profiles (average error below 1%, and up to 10% in the final cycles). Further, flow cell cluster densities were increased from 5-12 million clusters to about 35-60 million clusters per lane (and twice that for HiSeq instruments; where clusters on the top and bottom of the flow cell are read). A technical update made sequencing of the reverse strand of each molecule possible. Using this "paired-end sequencing" approach for determining the reverse strand, doubles the amount of sequence data generated. Known insert size, and thereby a known distance separating the paired reads obtained, provides additional information for later assembly or mapping [8]. This technical update of the Genome Analyzer in 2008, the Paired End (PE) module, also allowed the hybridization of further sequencing primers in the same strand orientation, making it possible to sequence a sample index (i.e. barcode) as part of the ligated adapter [9,10]. Such an index read allows for multiple samples to be sequenced in one lane (multiplexing). These can later be computationally separated based on the sequence of their index.

During progression of the sequencing run, or when images for all cycles have been collected (depending on the setup and version), the four images captured per tile are overlaid (registered) and light intensities are extracted for each cluster and cycle [1]. The resulting cluster position template is then aligned with images of all cycles and the intensities minus the surrounding background in the four different images extracted. Resulting intensity files serve as input for base calling - the conversion of intensity values into bases. Base calling on the Illumina platform is complicated by at least two effects: (1) a strong correlation of the A and C intensities as well as of the G and T intensities due to similar emission spectra of the fluorophores used and their limited separation by optical filters, and (2) dependence of the signal for a specific cycle on the signal of the cycles before and after, known as phasing and pre-phasing, respectively. Phasing and pre-phasing describe the loss of synchrony in the readout of the sequence copies of a cluster. Phasing is caused by incomplete removal of the 3' terminators and fluorophores as well as sequences in the cluster missing an incorporation cycle. Pre-phasing is caused by the incorporation of nucleotides without effective 3'-blocking. The proportion of sequences in each cluster which are affected by phasing and pre-phasing increases with cycle number; hampering correct base identification [11-14].

From this whole process, the Illumina user typically obtains sequences and per base quality scores. The set of sequences for each lane is usually quality filtered and the user gets a summary report for judging run quality. Finally, the Illumina CASAVA package provides additional tools and an interface to the visualization routines in Illumina's Genome Studio. Different commercial as well as free programs are available that replace some parts of the processing such as image analysis [15], base calling [11-15], quality assessment (e.g. TileQC [16] or FastQC [17]), mapping [18-21], as well as downstream data analysis and processing [8,22-25]. There is a large community of users and developers for the Illumina platform; the http://seqanswers.com website is an excellent resource when starting to explore the variety of programs available for analyzing the data generated.

## Results

We present each stage of a sequencing project from the generation of sequencing libraries to the base-calling of the sequences. For each step we discuss the potential effects on data analysis and final data quality. Where possible we offer suggestions and guidelines on how to avoid specific artifacts that arise during sequencing.

### Sequencing libraries, minimum insert size and adapter artifacts

The most important requirement for a DNA library to be sequenced on the Illumina platform is the presence of specific outer adapter sequences, complementary to the oligonucleotides on the flow cell used for cluster generation, the so-called "grafting sequences". As different sequencing primers can be used (see below), the rest of the library design is very flexible and various library preparation protocols with partially distinct adapter sequences are used for specific applications. Library adapters can be added by single strand ligation (e.g. Illumina small RNA protocol), double strand blunt-end ligation (e.g. for a multiplex protocol [10]), double strand overhang ligation (e.g. A-overhang for Illumina genomic library protocols, and restriction enzyme overhangs in the Illumina DGE protocols), or by extension from overhanging primers (e.g. multiplex PCR or molecular inversion probes [26,27]). Each of these approaches has a different susceptibility to the creation of library adapter dimers, chimeric sequences and other library artifacts. Each therefore requires a different approach to enrich for only those molecules with correctly added adapters, and to remove short/no insert molecules and molecules which are too long (> 800nt) from the library before sequencing. While short-insert molecules will, as described below, directly impact data analysis, longer molecules will perform differently in flow cell generation and generate more wide-spread and less dense clusters. If not accounted for by modified cluster generation protocols, these will result in lower quality reads.

Failure to perform an enrichment during library preparation has two potential effects: (i) the artifact sequences may have a negative impact on the image analysis and base-calling which are both challenged by an overrepresentation of one sequence population (see below) and (ii) sequencing of large numbers of such artifacts is uneconomical and lowers the potential number of informative sequences that can be generated per run. Libraries prepared from small amounts of input material tend to suffer from a higher fraction of library artifacts due to the relative abundance of adapter oligonucleotides compared to insert molecules.

**Figure 2:**
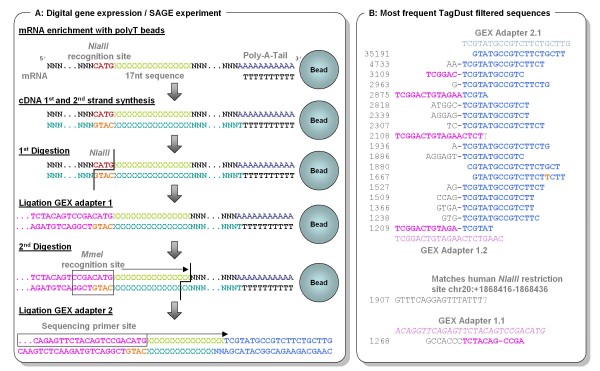
**Adaptors and adaptor chimeras are a common sources of sequence artifacts**. Specific outer adapter sequences, complementary to the grafting sequences on the flow cell are essentially the only requirement for sequencing a DNA library on the Genome Analyzer platform. As different sequencing primers can be used, library design is very flexible and various protocols with partially distinct adapter sequences have been established. The Illumina *NlaIII *DGE tag protocol illustrated here (a protocol for digital gene expression tag profiling) uses short adapters which are not compatible with paired end sequencing and are added by overhang ligation (A). For this protocol the majority of adapter dimers are removed by a gel excision step after library preparation. However, the protocol may also create adapter chimeras with a length comparable to the targeted library molecules. The resulting chimera sequences also show the sequences required for cluster generation as well as the necessary priming site, causing them to be sequenced together with the real DGE tags. A program like TagDust [29] can be used with the original adapter and primer oligonucleotide sequences to identify such artifacts (B). Shown are the twenty most frequent identified artifacts from one lane with human DGE tags, as well as the oligosequences they might be based on. One of the 20 sequences seems to be a real DGE tag that was incorrectly identified as an artifact.

It is possible to computationally post-process sequencing data in cases where enrichment has not been performed. Figure 2, exemplifies for the Illumina *NlaIII *DGE protocol (a protocol for digital gene expression tag profiling) that adapter chimeras might be created which are of comparable length as the targeted library molecules and thus may not be removed by selecting a specific library insert-size (e.g. by gel length selection, silica column purification or Solid Phase Reversible Immobilization (SPRI) purification [28]). In this case, a program like TagDust [29] can be used with the original adapter and primer oligonucleotide sequences to identify such artifacts in a library (Figure 2B). This program can be either used to directly remove these sequences or, for a representative lane, its results can be clustered and the most frequent ones used with other software tools.

Inappropriate size selection during library preparation may also complicate analysis due to partial sequencing of the adaptor at the sequence ends. Thus, when selecting for insert-size, it should be considered that current experimental methods generally do not provide precise length cutoffs. The lower cutoff selected should therefore be well-above the desired sequencing length. For sequence reads where part of the adapter sequence is included, the position in the sequence read at which the adapter sequence begins has to be identified and the read trimmed appropriately. Unfortunately, this is not part of the standard Illumina data processing and also non-trivial for short adapter fragments, especially given the increasing sequencing error at the end of reads. If reads are not filtered for known chimeras and trimmed for adapter sequences, these may interfere with mapping/alignment and reads will either be incorrectly excluded or placed incorrectly. In both cases downstream data analysis will be affected.

**Figure 3:**
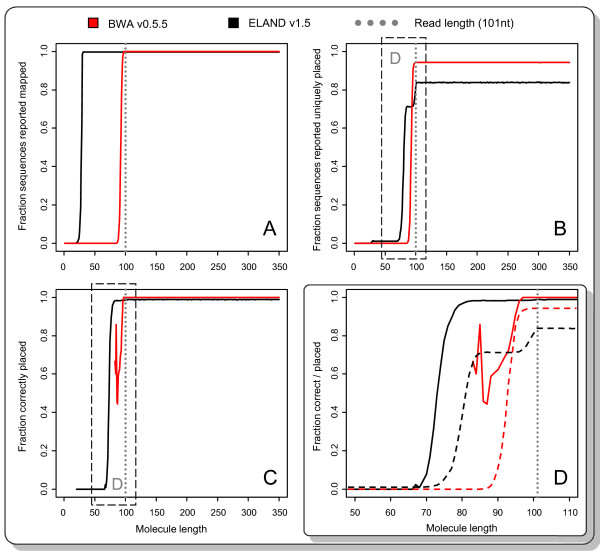
**Effects of adapter sequence inclusion on mapping**. Untrimmed adapter sequence at the read ends can interfere with alignment/mapping. We simulated 101-cycle human genomic shotgun reads for an Illumina Paired End library with 10,000 reads for every adapter starting point between 1 to 350nt, and the error profile observed for an actual run of this length. On this data set, we tested how ELAND and BWA are affected by inclusion of adapter sequence: (A) ELAND requires only a fixed seed (here 32nt) in the beginning of the read. Adapters beginning after this seed region may therefore have no effect on the output. ELAND reports 98% successful mappings for all simulated reads of at least 30nt insert size (2nt of adapter sequence being compensated by 2 mismatches allowed in the seed), BWA only reports 98% successful mappings for reads with an insert size of at least 97nt. (B) Frequently only uniquely placed molecules are considered in data analysis. ELAND reports the first uniquely placed fragment for 20nt insert size. BWA reports the first three uniquely placed fragments (mapping quality above 20) for an insert size of 83nt. (C) All uniquely placed reads reported by ELAND up to an insert length of 67nt are placed incorrectly (when comparing to the coordinates the sequence was extracted from), as is one of the 3 reported by BWA for an insert size of 83nt. When requiring 98% correct placements, ELAND handles up to 14nt of adapter (83nt insert size), while BWA can only compensate with mismatches for 4nt of adapter sequence (97nt insert size). (D) For analysis purposes, BWA shows the better performance due to the lower number of false positive placements. Moreover, for an insert size of at least the read length (i.e. no adapters interfering with the alignment), BWA reports 99.999% of uniquely placed reads (94.2% of all reported alignments) at the designated genomic positions, while ELAND only reports 98.757% of the uniquely placed reads (83.8% of all reported alignments) at the correct position.

In order to test how Illumina's ELAND mapper as well as the widely used mapping program BWA [20] are impacted by incipient adapter sequence, we simulated 101-cycle reads of an Illumina Paired End genomic library with 10,000 reads for every adapter start point between 1 to 350nt and the error profile observed for an actual run of this length (Figure 3). Considering that both mapping programs implement very different approaches (seed alignment versus semi-global alignment of the whole read respectively), the performance of Illumina's ELAND mapper is expected to be different from BWA. Since ELAND requires only a fixed seed in the beginning of the read (typically of 32nt length) adapters starting after this seed region should not affect ELAND's mapping. Indeed, ELAND maps 98% of all simulated reads of at least 30nt insert size (2nt of adapter sequence being compensated by 2 mismatches being allowed in the seed), while BWA only reports 98% successful mappings for reads with an insert size of at least 97nt. More relevant for many analyses, however, is the number of mappings reported to be uniquely placed and whether they are mapped at the correct position in the genome. ELAND reports a uniquely placed 20nt-insert-size read, but it is placed incorrectly (as are all uniquely placed reads reported up to an insert size of 67nt). BWA reports the first three uniquely placed fragments (mapping quality above 20) for an insert size of 83nt (2 of them are correctly placed). If we require that 98% of the reads are correctly placed, ELAND achieves this for insert sizes of 83nt and above (14nt of adapter), while BWA can only compensate with mismatches for 4nt of adapter sequence (97nt insert size). However, BWA provides a lower total number of false positive placements due to the inclusion of adapter sequence (8490 vs. 6308). Moreover, for an insert size of at the least read length, BWA reports 99.999% of uniquely placed reads (94.2% of all reported alignments) at the correct genomic positions, while ELAND only reports 98.757% of the uniquely placed reads (83.8% of all reported alignments) at the correct genome coordinates. BWA therefore provides a more accurate mapping of these reads for downstream analysis.

While length selection and dimer removal are important for the cost-effective sequencing of a library and downstream data analysis, experimental methods to achieve these generally consume sample material and may bias molecule representation. It is therefore often only practical to apply a minimum of these purification steps in order to maintain library quantity and complexity. In such cases, downstream sequence filtering prior to data analysis becomes extremely important.

### Short-insert libraries in paired-end sequencing experiments

When libraries containing inserts shorter than the sum of forward and reverse read cycles are created, these can be sequenced from both ends to obtain higher quality sequence information for the overlapping sequence part. For such paired-end reads the correct identification of the adapter is eased by maximizing autocorrelation of the two reads as well as requiring identical adapter start positions for both reads [30-32]. This strategy is more powerful than alignment(-like) approaches used for identifying adapter starts in single reads, which frequently remove sequence from the read ends that match the adapter by chance, or which do not identify real adapter sequence due to the higher sequencing error at the end of reads. Thus, for short insert libraries, paired end sequencing is preferable. As previously reported [30], the read merging performed for these short-insert libraries considerably decreases the number of errors and creates sequences reflecting the original outer molecule length (e.g. of interest for authenticity of ancient DNA samples [31]). Applying this merging approach to the simulated data set described above, but this time using both paired-end reads, we see a factor of 5 reduction in the error rate of all merged sequences (average error of 0.24% reduces to 0.05%; Additional File 1). For sequences shorter or equal to the read length a reduction by a factor of about 21 (0.146% to 0.007%) is observed.

### Library contamination

Sample contamination during library preparation from other DNA/RNA sources might be an important issue for some types of analyses and applications. Contamination may be introduced by the experimenter or may stem from lab chemicals and equipment. Library preparations starting from low amounts of sample DNA and protocols using single strand ligation procedures can be considered the most prone to contamination. While avoidance of contamination is the most desirable approach, it has been suggested (e.g. [33]) that reads can be filtered by the alignment to the putative contaminant sequence before data analysis. However, such filtering may introduce biases in the data, especially if sequences are short and/or the evolutionary distance between contaminant and sample is low. This is a frequent problem in ancient DNA studies of early modern humans, and Neandertals where contamination with even small amounts of modern DNA can quickly dominate sequencing output. Here the fraction of contamination can be deduced from informative sites (i.e. sites of known fixed differences between species/populations) and the fraction of contaminant molecules determined [31,32,34]. This ratio can then be used in statistical models during data analysis. If no informative sites are known, estimates of contamination may be obtained from biallelic or triallelic sites in haploid/diploid sequences. Hence, also the sex of the sample may be exploited by counting Y chromosomal alignments in female samples and determining × chromosomal heterozygosity for males [35,36].

Cross-contamination after library preparation (e.g. during preparation of the sequencing run) can be easily identified and filtered from the final sequencing data when sample-specific barcodes are included in the libraries and determined during sequencing [9,10]. A different problem may arise when pools of barcoded libraries are amplified simultaneously; in such experiments "jumping PCR" may cause barcodes to be transferred between samples [37-40]. High error in the sequencing, amplification or synthesis of barcodes, especially for those with limited sequence distance from one another, as well as barcode contamination during handling or mixed clusters/small physical cluster distance on the flow cell can lead to sample misidentification. In projects for which data analysis is susceptible to any of these types of contamination, appropriate measures have to be taken to minimize contamination and to estimate its impact on analysis results.

### Alternative sequencing primers

The sequencing primers used in sequencing of libraries constructed for different applications may differ. During flow cell preparation, different sequencing primers can be hybridized in each lane. However, in contrast to the first read prepared on the Cluster Station/cBot, it is not possible to use lane specific primers for the reverse read or for the index read prepared in the sequencing instrument. While it is technically possible to mix primers, this is generally not recommended due to potential mispriming. As a result, for paired-end as well as indexed sequencing runs the same library type has to be loaded on all eight lanes of the flow cell. Thus, while functional custom sequencing libraries are easily designed, it is generally not advisable to handle several library types and sequencing primers simultaneously due to the increased risk of sequencing primer mix-ups in the preparation process and the potentially long waiting times for runs with appropriate primer configurations when small-scale projects have to be sequenced on a shared flow cell. Further, when projects require a control lane or control read spike-in (see corresponding section below), the use of different adapter sequences/sequencing primers means that matching control libraries are also required.

### Machine adjustment and run preparation

The correct adjustment of the sequencing instrument is an important prerequisite for producing high quality sequencing data. Information on settings for each instrument and software version is available from the vendor's technical documentation. Individual instruments as well as sequencing kits and flow cells have variation. Therefore, correct instrument adjustment should be frequently checked and preparation of a sequencing run done with much attention to detail. Besides from attention to the liquids and optics of the instrument, the correct functioning of thermal elements and cooling devices are equally important for high quality runs.

**Figure 4:**
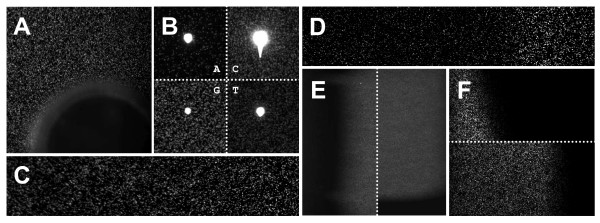
**Origin of image artifacts**. Correct instrument adjustment is an important prerequisite for producing high quality sequencing data. Preparation and start of a sequencing run has to be done with careful attention to avoid or identify the following instrumentation artifacts: (A) Air bubbles, caused by leaks, insufficient priming of reagent pumps and long waiting times. Bubbles can obscure parts of the images or reduce chemistry efficiency. (B) Particles in the sequencing chemistry (e.g. crystals from an unfiltered incorporation mix) frequently result in image artifacts. (C) Incorrect adjustment of stage flatness and stage tilt can cause distortions, i.e. parts of the image are sharp while the rest is out of focus. A similar effect limited to tiles at the flow cell edges, can originate from liquids covering the flow cell surface. (D) Reflections in the GA instrument can cause variation in cluster brightness, like the commonly observed band of bright clusters in column 2 of lane 8. (E) If the position of laser excitement is not in sync with imaging (footprint) on GA instrument, a black straight band can be observed at the edges of multiple tiles (partially with comb like slots). (F) If this effect is limited to tiles at the flow cell edges, oil coverage is insufficient.

When loading the chemistry and flow cell, all connectors should be checked for leaks, the correct priming of pumps with the reagents validated and long waiting times avoided. This is to prevent air bubbles in the pump, tubing and finally on the flow cell, which could cover parts of the images (Figure 4A) or reduce chemistry efficiency, due to smaller effective volumes and incomplete coverage of the inner flow cell surface. Leaks most frequently originate at the flow cell connectors (GA) or flow cell gaskets (HiSeq). While gaskets are replaced with each flow cell, the flow cell connectors of GA instruments may wear away or break. Loading reagents is simplified for HiSeq instruments, which store all reagents in two racks within the instrument - one for sequencing reagents and one for paired end (PE) reagents. Special funnel caps have to be used with the reagents to allow sippers to be lowered into the liquids. Bending of the sippers may cause reduced flow from the reagent. The HiSeq can only hold ten tubes for PE and multiplex chemistry (the GA holds up to 25 tubes). Thus, three of these ten positions are shared for PE preparation and multiplex chemistry on HiSeq, requiring more user interaction for multiplex runs and increasing the risk of reduced reagent flow and air in the instrument tubing.

**Figure 5:**
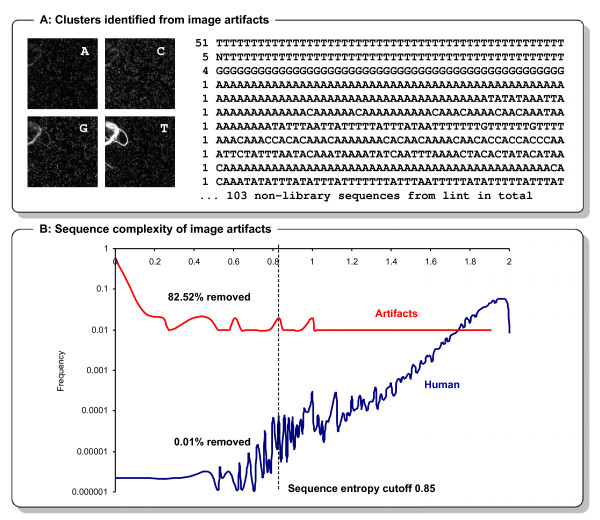
**Image artifacts can generate false sequences**. Cluster identification can identify crystals, dust and lint particles as well as other flow cell features as sequence clusters (A). Indicated are 103 non-library sequences originating from a lint particle that has been observed in a library that was sequenced with a three base pair tag ('GAC') in the beginning of each read. In this case, non-library sequences could therefore be distinguished based on these first three bases. The fraction of such artifact clusters is increased for low loading density and low intensity runs. A sequence entropy filter is efficient for removing the majority of these sequences (82.52% for a cutoff of 0.85), but also removes non-artifact sequences (B) - as indicated in the figure, 0.01% of the human reference genome (GRCh37/hg19). For 3'/5' tagged libraries or indexed sequencing libraries, filtering for the index/tag is therefore superior to base composition/sequence entropy filters for removing such sequencing artifacts.

In cases where several sequencing kits are required (typically more than 36 cycles for GA and 50 cycles for HiSeq), all sequencing kits required for sequencing reads on the same strand should be thoroughly mixed before splitting them by tube volume in order to prevent later problems with base calling due to different fluorophore intensities/chemistry performance. On GA and HiSeq, sequencing reagents can only be primed through the flow cell, while the PE/multiplex reagents can be primed using a separate priming pump. When refilling the sequencing reagent during the run, air bubbles in the hoses must be prevented as no additional priming can be performed. Further, the incorporation mix should be filtered and centrifuged before adding the polymerase and loading. This reduces chemistry crystals and lint artifacts on the images. Particles on the flow cell may result in excessively strong light signals (Figure 4B) covering large areas of the imaged flow cell. These may obscure actual clusters or cause cluster-like structures which are then detected during the cluster identification process (Figure 5A).

After performing the first base incorporation, the correct adjustment of the horizontal positioning (Z-value) of the flow cell stage to the focus layer(s) as well as the flow cell tilt should be checked with a first cycle report before the actual first cycle imaging. For Genome Analyzer instruments, the complete illumination of tiles (footprint and oil application), the adjustment of the focus laser, the maximum focus range and stage tilt should be checked in addition. Some of the necessary adjustments can be done directly by a skilled lab technician, if a problem is correctly identified.

Problems with the stage flatness can be identified if image distortions, i.e. only part of the imaged tile is sharp while the rest seems out of focus (Figure 4C), are seen on multiple tiles and not limited to the edges of the flow cell. If this effect is limited to tiles at the flow cell edges, liquids (including oil in the case of a GA instrument) covering the flow cell surface could be a more reasonable explanation. While in the first case the adjustment of the instrument stage by an Illumina technician is required, in the latter case, the flow cell has to be removed, cleaned and reinserted into the instrument (otherwise the liquid will be spread to more tiles over the course of the run). Commonly, a band of brighter clusters is observed on the right side in the second track of lane 8 for GA instruments (Figure 4D). This is probably caused by a reflection of laser light on the right flow cell edge and does not appear to cause problems for data analyses.

Flow cell tilt is measured automatically with current software versions; if a too high value is determined, a wrong alignment of the flow cell in the instrument is a likely source. However, an uneven outer flow cell edge may also cause this problem; in this case, the flow cell tilt has to be manually set by averaging out the uneven edge. In case the tilt is set incorrectly, tiles will not be positioned correctly within the lane boundaries. If a black straight band is observed for a GA instrument on one of the four edges of multiple tiles (partially comb-like slots can be observed, Figure 4E) the position of laser excitement may not be in complete synchronization with imaging (footprint). In this case the laser spot can be corrected by two adjustable screws in the GA instrument. If this effect is limited to tiles at the flow cell edges, oil coverage below the flow cell is not sufficient and additional oil has to be applied (Figure 4F).

GA focus calibration reports should be checked for continuous × and Y values. Discontinuities in these values are frequently caused by confusion of the focus laser spot with its reflection and, like error messages of the spot being close to the image edge, these are the result of an incorrect focus laser adjustment. If the focus laser is not readjusted by an Illumina technician, incorrectly focused tiles will be obtained. High values for the maximum focus range in the first cycle report might hint at incorrect flow cell alignment which causes bending of the flow cell. In this case, Z-values typically decrease considerably from the middle of the flow cell towards both ends. However, if Z-values decrease or increase monotonically from top to bottom of the flow cell the stage's tilt can be adjusted on GA instruments using precision mechanics screws. If not readjusted, the maximum focus range will be exceeded during sequencing and incorrectly focused clusters will result.

If low intensities are observed in the first cycle report (e.g. due to long handling times), the primer hybridization should be repeated. This step can be performed with a sodium hydroxide wash and a hybridization protocol either in a Cluster Station/cbot or with a PE module/HiSeq instrument directly in the instrument, as long as identical sequencing primers are being used for all lanes. The typical intensity range is highly dependent on the exact chemistry and image analysis software version and is available from the corresponding vendor documentation.

It is advisable to temporarily store run images (only possible for the Genome Analyzers) or to permanently store reduced size thumbnails of the run image (possible for all platforms). When problems with a run are observed direct observation of images frequently provides very useful information for troubleshooting.

### Image analysis

The images for each of the four fluorophores, for all tiles per lane in each cycle have to be overlaid (registered) and the light intensities extracted for each cluster and cycle. In cases where all reads, or a vast majority of the reads, have identical initial sequences, the image analysis software removes clusters as it considers these to originate from one initial DNA molecule and therefore uninformative. This reduces the overall yield (Additional File 2) from a sequencing run by 10% - 30% depending on the loading density and software version. Libraries produced from restriction-digested molecules or libraries with tag/barcode sequences added on the outer edge are frequently affected in this way. Starting the off-instrument image analysis (using the built-in Firecrest module) with a user-defined *nr *parameter, which sets the number of cycles used for cluster identification, can be used as a work-a-round. This can only be achieved in cases where it is possible to save the full run images off the instrument. The default value of this parameter depends on the analysis pipeline version (below v1.3: 1 and not configurable; v1.3 to v1.5: 2 cycles, v1.6: 4 cycles, v1.8: 5 cycles). However, the design of project specific primers should be preferred over changing image analysis parameters, as the data transfer and offline analysis of images requires an additional investment in money and time. It is possible to change the number of detection cycles used by the instrument software (RTA). This requires additional disk space for on-instrument storage, and will increase the waiting time for calculation of the cluster position template. Changing the number of cycles used for cluster identification may also increase the fraction of artifacts incorrectly identified as clusters (see sequencing artifacts section). Further, when the majority of sequences are identical in the first cycles, problems with base calling may occur (see base calling section).

The optimal number of clusters per mm^2 ^of the lane largely varies depending on the instrument version and the library being sequenced. With the software version RTA/OLB v1.8, a complex library without base composition biases can be loaded with 600,000-700,000 per mm^2^, while a low complexity library should be loaded in the range of 500,000-600,000 per mm^2^. Precise values for each instrument and software version should be obtained from the vendor's technical support. Differences between low and high complexity libraries, are caused by an increased background signal (lower base qualities) and cluster tracking issues (N bases) which occur when many reads have the same base in a cycle and thus end up on the same image. If cluster densities are reduced, the background signal from clusters in close proximity is reduced which leads to higher intensity over noise values being measured. Loading the correct amount of library DNA to obtain these designated cluster counts requires a precise quantification by qPCR or high-resolution chip-based capillary electrophoresis [41]. A stable and accurate library quantification procedure is therefore also one of the main prerequisites for performing high-quality sequencing runs.

### Sequencing artifacts and sequence quality

The random dispersion cluster generation process performed allows for high loading densities but also complicates the identification of cluster positions from images. Image analysis and cluster identification algorithms used for this purpose often identify sequencing chemistry crystals, dust and lint particles as well as other flow cell features as sequence clusters (Figure 5A). The fraction of such artifact clusters is increased for low cluster densities (as the number of these artifacts does not necessarily increase with cluster density) and for low intensity runs. Low cluster intensities may have multiple causes including: (1) reduced cluster growth during bridge amplification, (2) clusters which are widespread (e.g. due to large library insert sizes), (3) inefficient sequencing primer hybridization, (4) degraded/bleached fluorophores, poor performance of the polymerases due to production/storage, handling issues or bad scanning buffer, or (5) increased background signal. When low intensities are observed for the first time, primer hybridization and first base incorporation should be repeated to exclude the most frequent causes.

If the cluster identification software identifies sequencing chemistry crystals, dust and lint particles as clusters, the resulting sequences are typically of low sequence complexity (i.e. consisting of long stretches of identical bases; Figure 5A) and may be partially also of low quality (Additional File 3). However, such sequences are typically not completely removed by signal purity/quality filters. Further, freely moving crystals/lint may also appear in different positions in later cycles and obscure the signal of regular clusters. Depending on their size, these may even cover a larger fraction of a tile and thereby prohibit the correct read out of many clusters in one or several cycles (Figure 4B). In combination with air bubbles (Figure 4A; caused by leaks or depletion of some reagent), these impermanent features are a frequent source of missing base calls (Ns) and sequencing error. The number of these fixed and movable artifacts can be reduced by a clean sequencing set-up and the steps described above. In extreme cases, the exclusion of complete tiles from analysis during post-processing should be considered [16].

The low sequence complexity of reads from most of these mis-identified artifacts can be exploited by a sequence entropy filter or another base composition/base frequency filter. For indexed sequencing libraries [9,10], such clusters are efficiently removed by an index sequence filter step. The same applies for libraries with tag sequences. Filtering for index and tag sequences should be considered superior, as other filters may also remove non-artifact sequences of low complexity (Figure 5B).

Further, random cluster dispersal results in a wide range of inter-cluster distances, causing different susceptibility of individual clusters to neighboring signals. At the most extreme two distinct sequence populations can be completely merged in one mixed cluster, resulting in the detection of a mixture of signals from the different sequences. Depending on the ratio of the two sequences and their sequence similarity, the resulting sequence can be close to random with low overall base quality scores, or may be more similar to one of the original molecules and show low base quality scores/higher error rate for only some positions in the read. This effect of cluster distance on signal purity causes sequencing errors to be non-randomly distributed, i.e. the fraction of reads with two errors is not equal to the squared fraction of reads with one error, but is considerably higher (Additional File 4). Hence, some clusters accumulate error disproportionately due to their close proximity to another sequence cluster. Such clusters can be identified by a high frequency of low quality bases. The default Illumina signal purity filter called 'chastity' requires that for the first twelve cycles (in later versions of the analysis pipeline the first 25 cycles, allowing one outlier) corrected intensities for the bases called are 1.5 times higher than the next highest base intensity. However, a simple quality-score-based filter (which is by design highly correlated with signal purity) applied over all reads and not only the first bases of the run is preferable. We have found a lower base quality score cutoff of 15 and allowing one outlier every 20 cycles to be an effective filter.

### Standard base calling and sequence composition

The Illumina base caller uses a model-based approach for the conversion of intensity values into bases. The run-specific parameters of this model (so-called cross-talk matrix and phasing/pre-phasing values) are determined from the first few cycles of each read. The cross-talk matrix is typically estimated from cycle 2, phasing and pre-phasing values from the first 20 cycles. This estimate is often incorrect when libraries have an unbalanced base composition in this part of the read. Such unbalanced base composition is common when a restriction site or some tag sequence is present in either the forward or the reverse read (in case of paired-end sequencing). The only read type for which this parameter estimation is not used is the index read. For the index, parameters calculated for the preceding read are applied. Therefore, for at least one lane in each run the base composition should be balanced over the thousands of clusters per tile, or a separate control lane has to be sequenced for estimating these base-calling parameters.

This control lane library is not limited to the commonly used φ × 174 genome; however the choice should be limited to a high complexity shot-gun library from an organism with close to 50% GC content, to account for assumptions in the parameter estimation process [1,13,14]. A genomic shot-gun library from most species can be used for this purpose. mRNA sequencing libraries have been suggested as a valid replacement for the control lane, however mRNA libraries (prepared from the standard Illumina-protocol using double strand ligation protocol) show a biased base composition in the first twelve bases of the reads [42] and are therefore no longer recommended.

### Quality control, read spike-in and alternative base callers

In addition to the estimation of base-calling parameters, a control lane also provides useful statistics for sequencing quality. For this purpose the addition of a spike-in φ × 174 sequences in every lane is highly recommended for runs on the Illumina platform, even in cases where a high complexity shot-gun library lane is available for parameter estimation. Given a known high quality control library, the obtained per lane statistics for this library can then be compared, even between different runs. The choice of φ × 174 as a quality control is again arbitrary; however a control sample should have the following features: (1) small genome with no similarity to the actual library sequenced, (2) completely known genome sequence of the exact sample to determine error development and (3) high complexity and balanced base composition to study error patterns. If condition (1) is fulfilled, the spike-in can be performed without the need for multiplex sequencing in order to later separate the control reads from other library molecules. The inclusion of φX spike-in in each lane rather than in a separate control lane is also a vendor-recommended procedure for HiSeq sequencing.

A fraction of less than 1% control reads is sufficient for creating these quality statistics for all lanes and even facilitates the use of a reference-based base calling approach, such as AltaCyclic [11] and Ibis [14], which increases the base calling accuracy. Despite differences in sequencing chemistry, run parameters and overall run quality, the application of an alternative base caller typically yields a reduction in error rate of about 20% (Additional File 5). When turning off automatic parameter estimation for the Illumina base caller and using default values from runs of comparable sequencing chemistry, this low fraction of control reads in combination with an alternative base caller allows for the omission of a dedicated control lane for libraries with unbalanced base composition.

Frequently projects use sequence data generated on different sequencing platforms, with varying versions of the sequencing chemistry and instrument software, or data produced in different facilities. This creates a need for assuring data quality and consistency. Quality score recalibration based on sample alignments to a reference genome has been identified as one solution to this problem [43]. Currently the most widely applied algorithm is part of BROAD Institute's Genome Analysis Toolkit (GATK) [44]. However, quality score calibration based on alignments with some divergence to the actual sample is problematic, especially if the divergence to the reference varies between samples. This could cause a biased correction of quality scores and fewer SNP calls for the samples with higher divergence (which is why the tool in GATK allows for masking of known SNP positions). If the quality scores calibrated on a reference alignment are used in an inter-species comparison and genome quality or species diversity varies (as is commonly the case), then qualities obtained from such calibrations will have a species bias (i.e. scores will be lower for the species with lower reference genome quality or higher diversity). Therefore, only spike-in control reads should be used for calibration of quality scores (as is implicitly the case for the Ibis base caller [14]).

## Conclusions

The Illumina platform provides users with the ability to construct application-specific sequencing libraries using a variety of lab protocols and possibly different sequencing primers. These libraries may differ in the observed artifacts like adapter dimers and chimeras, the susceptibility to contamination from external DNA/RNA sources as well as the ability of establishing a designated library insert size.

What is perhaps not widely realized is that the Illumina software does not handle artifact sequences, nor does it filter or trim adapters. Thus, some fraction of insert-adapter-chimeras or pure adapter dimers may end up in the final data analysis; where they may cause false alignments or, due to unsuccessful alignment, exclude short-insert-size molecules from analysis. Even when explicitly included, the identification of adapter sequence and adapter chimeras is not trivial. It is hampered by reads where only a few bases of the adapter are read and by the higher error rates at the end of reads. For paired end reads the correct identification of adapters is assisted by maximizing autocorrelation of the two reads and expecting equal adapter start points. For short insert libraries, paired end sequencing is therefore preferable. Further, the merging performed for short insert libraries allows for considerably reduced error rates in the paired-end-consensus sequences.

In addition to creating a high-quality sequencing library and quantifying it in order to calculate the correct loading density, the correct adjustment of the sequencer, handling, air bubbles as well as particles in the sequencing chemistry have a considerable impact on the quality of a run. These instrument adjustments are greatly simplified on the HiSeq instrument, however, on the earlier Genome Analyzers reflections, uneven application of oil and an imperfectly adjusted machine cause problems in data quality. Particles like chemistry crystals, dust and lint can cause pseudo sequence signals which then result in the inclusion of reads which do not correspond to molecules from the sequenced library. Tagging or indexing provides users with the ability to filter reads for bona fide library molecules and should be preferred over sequence complexity based methods which efficiently remove most of these artifact sequences, but which may introduce a bias due to the removal of real low complexity sequences.

Indices/tags placed in the beginning of reads may reduce sequencing throughput and increase error due to problems introduced in image data analysis and base calling. Performed as separate reads [9,10], as intended by Illumina, the error profile of the actual reads is not altered and multiplexing allows for the optimal usage of the increasing sequencing throughput.

Spike-in controls included in all lanes facilitates the assessment of run quality for individual lanes and whole runs. We suggest that improved base callers should be considered to obtain sequences of increased quality and that the PHRED-like base quality scores should be used for quality-based filtering based on the complete read rather than only on the beginning of the read. Quality score based filters are equally suited for filtering clusters accumulating error due to their close proximity to other sequence clusters and may also remove reads affected by freely moving artifacts in later sequencing cycles.

We summarize the most important principles as follows: (1) Filter all Illumina-supplied buffers and perform pre-run checks for leaks and instrument adjustment. (2) Check quality statistics as well as images for artifacts and the correct adjustment of the machine. (3) Filter sequence data for library artifacts like adapters and chimeras and examine the data for traces of contamination. (4) Remove artificial clusters by filtering for sequence complexity or, if possible, filter for tags/indexes. (5) Filter low quality reads based on quality scores of complete sequences. (6) Use alternative base callers to obtain the maximum yield of high quality sequences from a run. These simple guidelines enable the identification and elimination of most of the problems commonly encountered in sequencing runs done on the Illumina sequencing systems.

## Methods

### Simulated data

Simulated reads were obtained by extracting ten thousand 350nt long sequences, not containing N characters, from all chromosomes/contigs of at least 1 Mb in the human hg19/GRCh37 assembly. These sequences were then trimmed for the different molecule lengths/adapter start positions and paired end reads created. For sequences below the read length of 101, the forward and the reverse read adapter sequences were added (forward: AGATCGGAAGAGCGGTTCAGCAG GAATGCCGAGACCGATCTCGTATGCCGTCTTCTGCTTG, reverse: AGATCGGAAGAGC GGTTCAGCAGGAATGCCGAGACCGATCTCGTATGCCGTCTTCTGCTTG) and if required extended by further A bases at the end. On the resulting 3.5 million reads, the error profile extracted from the control reads of a 2 × 101 cycle version 4 sequencing chemistry run was applied by randomly mutating bases at the observed rate for each position and using two different approaches for simulating quality scores: (1) the quality score was set to the average error observed for the specific base-type in this cycle (i.e. all Adenines at the same position in the read have the same quality score) or (2) error-informative quality scores were created by adding a random number between 0 and 10 (uniform sampling) to the average quality score of this base when the correct base was simulated and subtracting a random number between 0 and 10 (uniform sampling) when a wrong base was simulated.

### Merging of paired end reads

The two reads from each cluster were merged providing the adapter sequences and requiring at least an 11nt overlap between the two reads. Reads were merged by sliding the reverse complement sequence of the reverse read along the forward read and determining the quality score adjusted sequence identity of forward and reverse read for the different adapter start positions. Reads were merged if the highest observed sequence identity in the read overlap was at least 90%. In the overlapping sequence, quality scores were combined (assuming equal likelihood for non-observed bases) and the base with the highest base quality score was called.

## Supplementary Material

Additional File 1 **Merging of paired end reads efficiently removes adapter sequence for short insert libraries and increases read accuracy**. Shown is the average sequencing error of the two simulated raw reads (black) in comparison to the sequencing error remaining after read merging for different adapter start points. The development is shown for two different types of simulated quality scores (red and green). In red, the quality score is the average error observed for the specific base-type in this cycle (i.e. all Adenines at this position in the read have the same quality score), while in green an error-informative quality score was simulated. For this type of quality score a random number between 0 and 10 (uniform sampling) was added to the average quality score of this base when the correct base was simulated and a random number between 0 and 10 (uniform sampling) was subtracted if a wrong base was simulated. The average reduction of error (starting from 0.244%) is 1.93 × (0.126%) for the position-dependent quality scores and 4.98 × (0.049%) for the error-informative quality scores. For sequences shorter or equal to read length (5-101nt) a reduction of error (0.146%) by a factor of 1.62 × (0.090%) and 20.88 × (0.007%) is observed, respectively. Sequences are required to have more than 10nt overlap for merging and merged sequences below 5nt are discarded as adapter dimers by the program. Click here for file

Additional File 2 **Reduction of the number of clusters identified in tile images due to identical tag sequences**. If all or the vast majority sequences start identical in the first read of a sequencing run, image analysis will consider a higher fraction of the clusters as being grown into each other and remove them. This effect is for example observed if libraries are made from restriction digested molecules or if tag/barcode sequences are added on the outer molecule edges and read in the first read. Changing parameters for an image offline analysis (Firecrest module) can be used as a work-a-round. The figure table shows cluster counts as well as a section of the image of the same tile in cycle 1 and 4 for a run from the Neandertal Genome project (Green et al: Science 2010) 080902_BIOLAB29_Run_PE51_1 in which the tag 'GAC' was read in the beginning of the first read. Cluster counts were obtained from IPAR v1.01 image analysis (cluster identification based only on the first cycle of the run) and the results for a version of the Firecrest v1.9.5 algorithm, in which cluster identification was done in cycle 4. Click here for file

Additional File 3 **Quality score distribution of artifact reads largely overlaps with the quality score distribution of regular reads**. Sequences resulting from crystals, dust and lint particles as well as other flow cell features are typically of low complexity (Additional File 2) but only partially of low quality. Plotted is the quality score frequency distribution (PHRED-scale, Ibis base caller) for all reads matching the 'GAC' library tag in the beginning of the read (black, n = 557,466,159 bases from 10,930,709 reads) as well as all sequences not matching the tag sequence and its one base pair substitutions (red, n = 3,481,668 bases from 68,268 reads). The data was obtained from lane 5 of the 080902_BIOLAB29_Run PE51_1 run from the Neandertal Genome project (Green et al: Science 2010). Click here for file

Additional File 4 **Non-random distribution of sequencing error across sequencing clusters**. Random cluster generation results in a wide range of inter-cluster distances, causing sequencing error to be non-randomly distributed across clusters. The fraction of reads with two errors is not equal to the squared fraction of reads with one error. Shown are the observed rates for reads with 1 to 5 errors for different Illumina Genome Analyzer data sets (solid lines) presented as test data sets for the Ibis base caller (Kircher et al: Genome Biology 2009) and the expected rates when extrapolating from the fraction of molecules with one error (dashed line). Click here for file

Additional File 5 **Reduction in sequencing error when using the Ibis base caller for different instrument chemistries**. Alternative base callers significantly reduce error rate and thereby increase the output of usable reads. The Ibis base caller (Kircher et al. Genome Biology 2009) has a wide support for different instrument and software versions, as well as for single read, paired-end read and multiplex sequencing runs. It is based on training sequencing cycle-specific machine learning models from a training data set, like for example a φ × 174 spike-in control. Based on this data, also quality scores are adjusted for each run and are therefore comparable between sequencing runs and libraries without further normalization. Click here for file
