# Supplementary figures and images for: Addressing challenges in the production and analysis of illumina sequencing data
Source: BMC Genomics. 2011 Jul 29;12:382. doi: 10.1186/1471-2164-12-382 (PMC3163567; doi:10.1186/1471-2164-12-382)

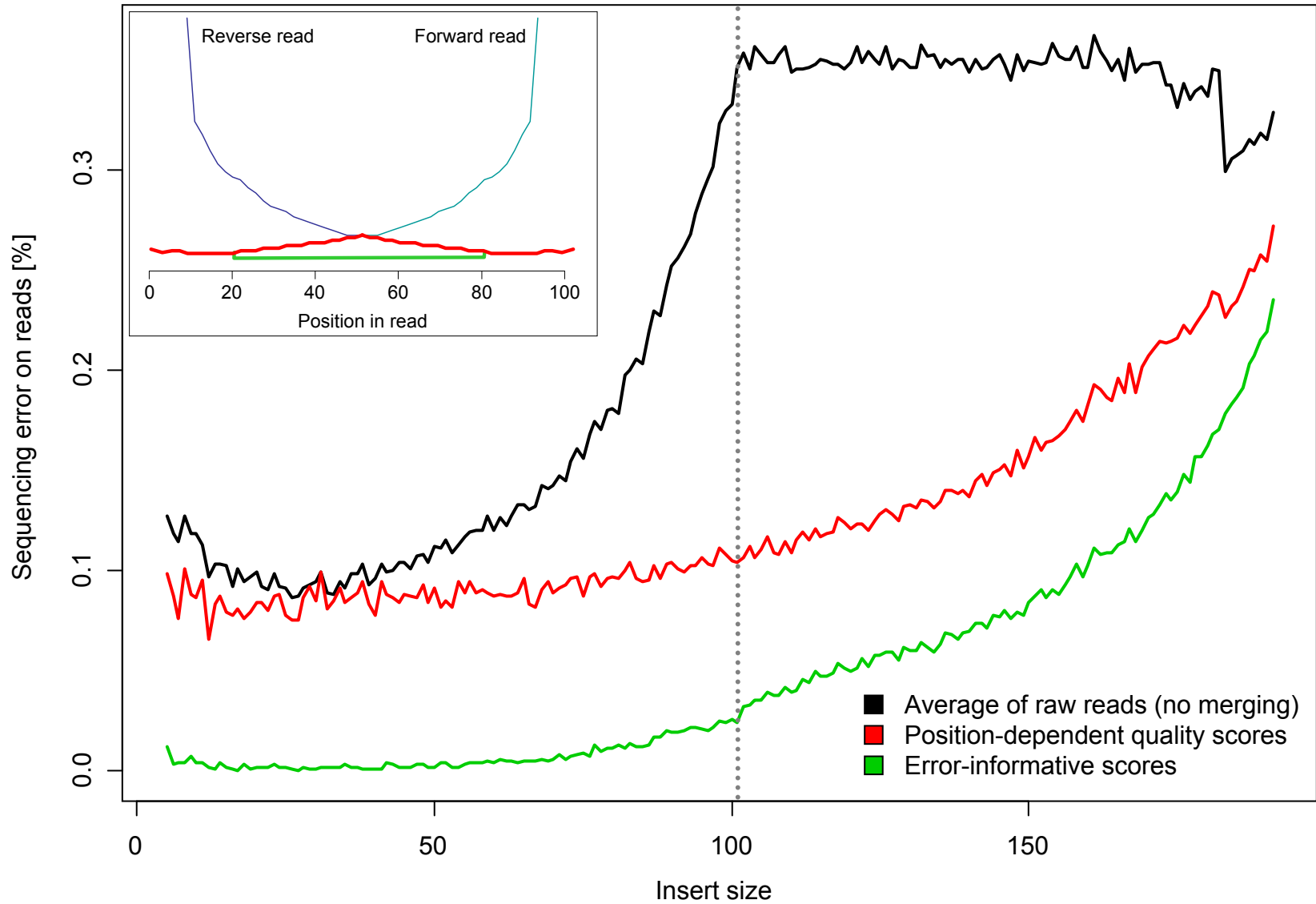

Supplement: Additional File 1 — Merging of paired end reads efficiently removes adapter sequence for short insert libraries and increases read accuracy. Shown is the average sequencing error of the two simulated raw reads (black) in comparison to the sequencing error remaining after read merging for different adapter start points. The development is shown for two different types of simulated quality scores (red and green). In red, the quality score is the average error observed for the specific base-type in this cycle (i.e. all Adenines at this position in the read have the same quality score), while in green an error-informative quality score was simulated. For this type of quality score a random number between 0 and 10 (uniform sampling) was added to the average quality score of this base when the correct base was simulated and a random number between 0 and 10 (uniform sampling) was subtracted if a wrong base was simulated. The average reduction of error (starting from 0.244%) is 1.93 × (0.126%) for the position-dependent quality scores and 4.98 × (0.049%) for the error-informative quality scores. For sequences shorter or equal to read length (5-101nt) a reduction of error (0.146%) by a factor of 1.62 × (0.090%) and 20.88 × (0.007%) is observed, respectively. Sequences are required to have more than 10nt overlap for merging and merged sequences below 5nt are discarded as adapter dimers by the program. [file 1471-2164-12-382-S1.PDF]

Quality score frequency distribution for library and non-library sequences

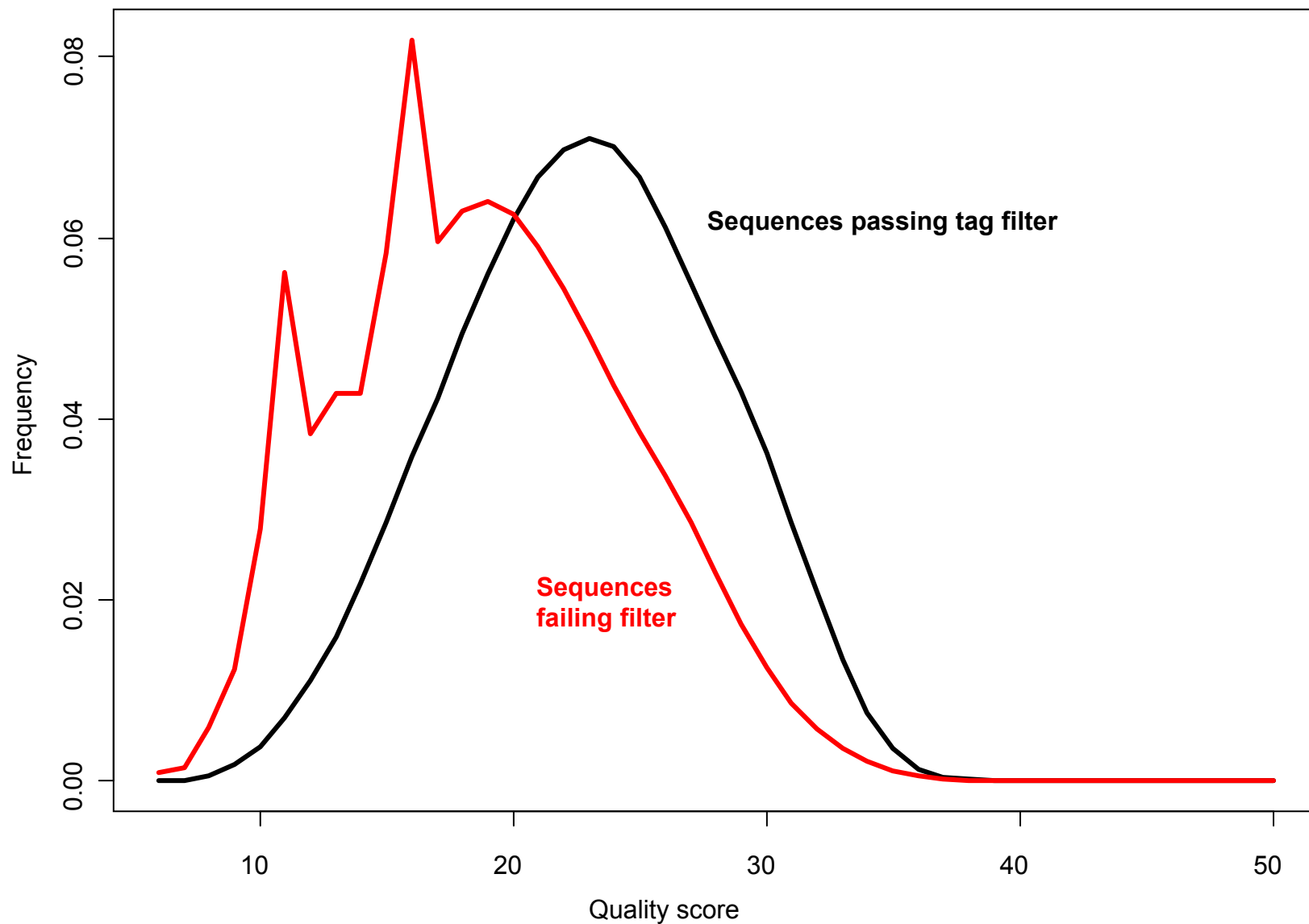

Supplement: Additional File 3 — Quality score distribution of artifact reads largely overlaps with the quality score distribution of regular reads. Sequences resulting from crystals, dust and lint particles as well as other flow cell features are typically of low complexity (Additional File 2) but only partially of low quality. Plotted is the quality score frequency distribution (PHRED-scale, Ibis base caller) for all reads matching the 'GAC' library tag in the beginning of the read (black, n = 557,466,159 bases from 10,930,709 reads) as well as all sequences not matching the tag sequence and its one base pair substitutions (red, n = 3,481,668 bases from 68,268 reads). The data was obtained from lane 5 of the 080902_BIOLAB29_Run PE51_1 run from the Neandertal Genome project (Green et al: Science 2010). [file 1471-2164-12-382-S3.PDF]

Frequency of molecules with number of errors

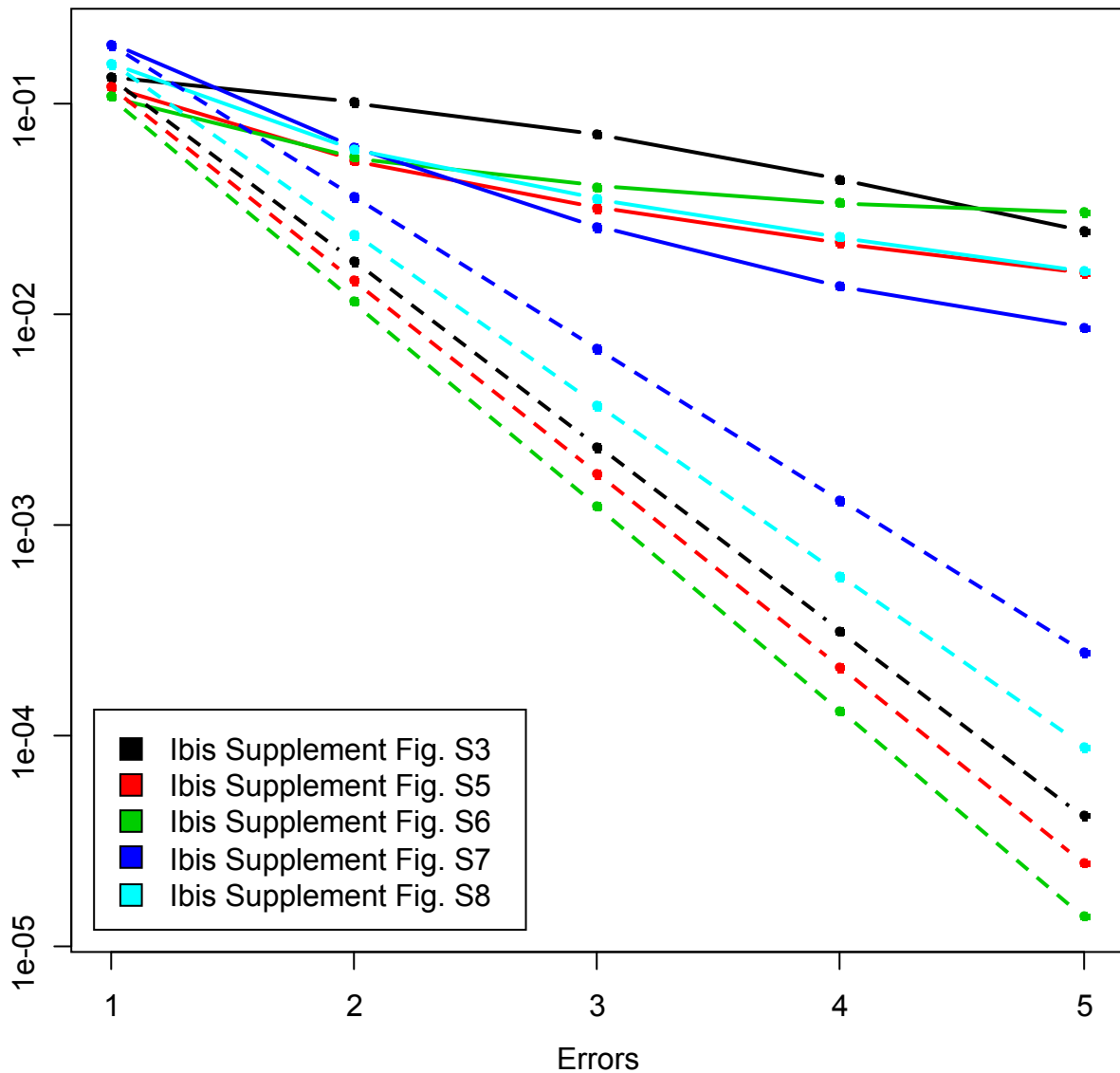

Supplement: Additional File 4 — Non-random distribution of sequencing error across sequencing clusters. Random cluster generation results in a wide range of inter-cluster distances, causing sequencing error to be non-randomly distributed across clusters. The fraction of reads with two errors is not equal to the squared fraction of reads with one error. Shown are the observed rates for reads with 1 to 5 errors for different Illumina Genome Analyzer data sets (solid lines) presented as test data sets for the Ibis base caller (Kircher et al: Genome Biology 2009) and the expected rates when extrapolating from the fraction of molecules with one error (dashed line). [file 1471-2164-12-382-S4.PDF]
