# Supplementary material for: Addressing challenges in the production and analysis of illumina sequencing data
Source: BMC Genomics. 2011 Jul 29;12:382. doi: 10.1186/1471-2164-12-382 (PMC3163567; doi:10.1186/1471-2164-12-382)

Lane	Sample	Conc. [pM]	1st Cycle	4th Cycle	Ratio
1	SL7	1.0	6,941,328	7,238,465	96%
2	SL7	2.0	8,871,763	12,646,584	70%
3	SL6	1.0	7,675,074	8,123,010	94%
5	SL6	1.5	9,061,321	11,200,209	81%
6	SL6	2.0	9,190,638	14,167,561	65%
7	SL8	1.0	6,773,787	6,834,065	99%
8	SL8	2.0	9,003,716	11,369,083	79%
4	PhiX	2.0	11,279,178	11,478,043	98%

G channel, 1st cycle

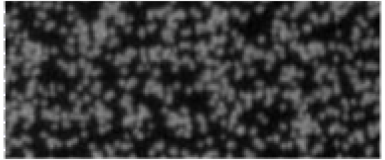

G channel, 4th cycle

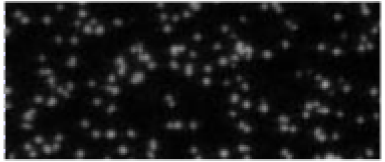

Supplement: Additional File 2 — Reduction of the number of clusters identified in tile images due to identical tag sequences. If all or the vast majority sequences start identical in the first read of a sequencing run, image analysis will consider a higher fraction of the clusters as being grown into each other and remove them. This effect is for example observed if libraries are made from restriction digested molecules or if tag/barcode sequences are added on the outer molecule edges and read in the first read. Changing parameters for an image offline analysis (Firecrest module) can be used as a work-a-round. The figure table shows cluster counts as well as a section of the image of the same tile in cycle 1 and 4 for a run from the Neandertal Genome project (Green et al: Science 2010) 080902_BIOLAB29_Run_PE51_1 in which the tag 'GAC' was read in the beginning of the first read. Cluster counts were obtained from IPAR v1.01 image analysis (cluster identification based only on the first cycle of the run) and the results for a version of the Firecrest v1.9.5 algorithm, in which cluster identification was done in cycle 4. [file 1471-2164-12-382-S2.PDF]
