# Supplementary material for: Addressing challenges in the production and analysis of illumina sequencing data
Source: BMC Genomics. 2011 Jul 29;12:382. doi: 10.1186/1471-2164-12-382 (PMC3163567; doi:10.1186/1471-2164-12-382)

| | | GA I (26nt) | GA II (51nt, v1) | GA II (77nt, v2) | GA II (76nt, v3) | GA IIx (101nt, v4) | GA IIx (101nt, v5) |
|---|---|---|---|---|---|---|---|
| ***Bustard*** | Perfect | 11.3% | 39.8% | 9.19% | 51.52% | 62.60% | 77.74% |
| | **Error** | **7.1%** | **2.0%** | **2.74%** | **0.89%** | **0.41%** | **0.36%** |
| ***IBIS*** | Perfect | 23.4% | 60.2% | 36.58% | 58.90% | 65.05% | 84.66% |
| | **Error** | **5.4%** | **1.1%** | **0.73%** | **0.65%** | **0.33%** | **0.23%** |

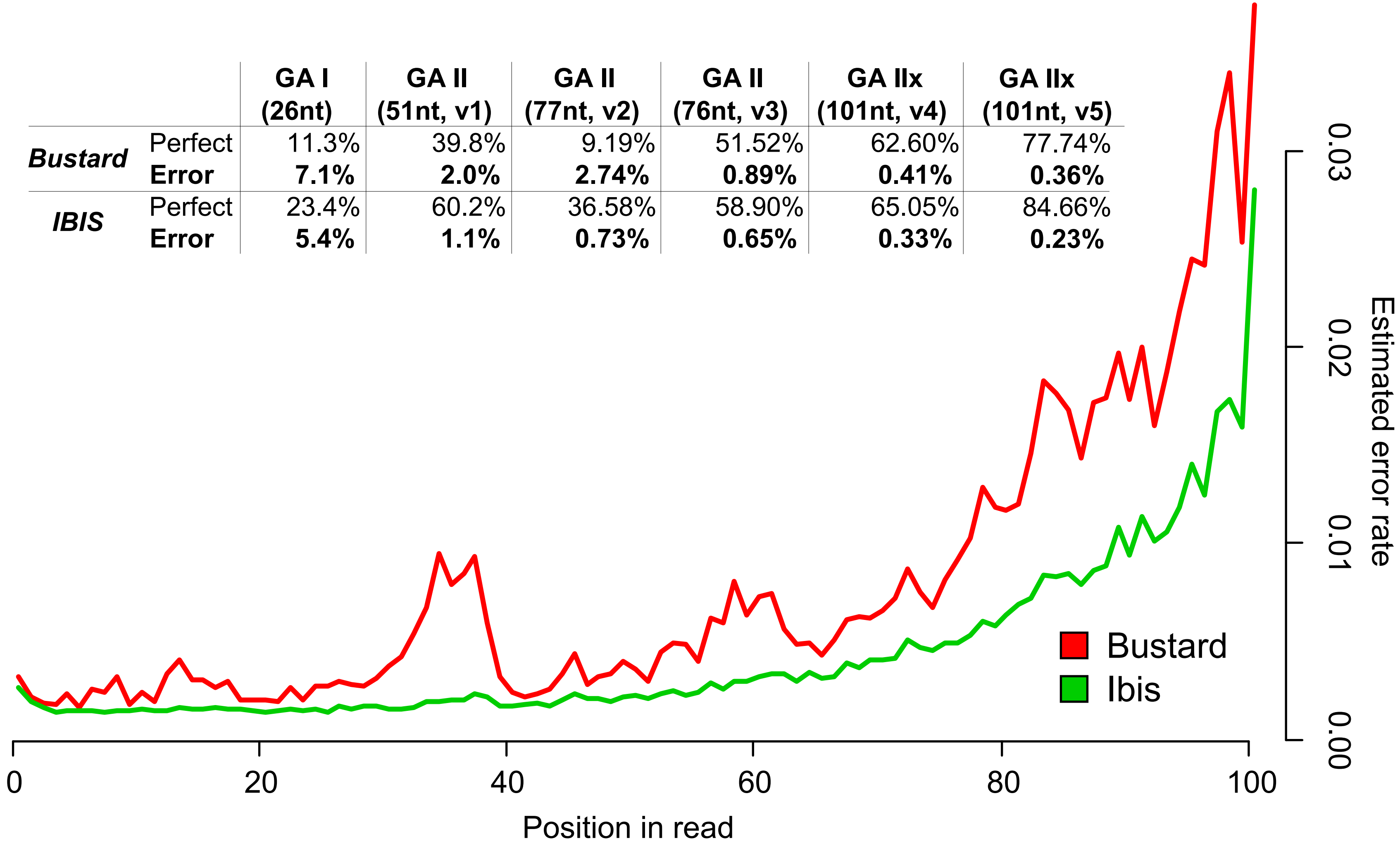

Supplement: Additional File 5 — Reduction in sequencing error when using the Ibis base caller for different instrument chemistries. Alternative base callers significantly reduce error rate and thereby increase the output of usable reads. The Ibis base caller (Kircher et al. Genome Biology 2009) has a wide support for different instrument and software versions, as well as for single read, paired-end read and multiplex sequencing runs. It is based on training sequencing cycle-specific machine learning models from a training data set, like for example a φ × 174 spike-in control. Based on this data, also quality scores are adjusted for each run and are therefore comparable between sequencing runs and libraries without further normalization. [file 1471-2164-12-382-S5.PDF]
